# Opioid Treatment Is Associated with Recurrent Healthcare Visits, Increased Side Effects, and Pain

**DOI:** 10.5811/westjem.18380

**Published:** 2024-09-24

**Authors:** Caroline E. Freiermuth, Jenny A. Foster, Pratik Manandhar, Evangeline Arulraja, Alaattin Erkanli, Charles V. Pollack, Stephanie A. Eucker

**Affiliations:** * University of Cincinnati, Department of Emergency Medicine, Cincinnati, Ohio; † University of Cincinnati College of Medicine, Center for Addiction Research, Cincinnati, Ohio; ‡ Duke University School of Medicine, Durham, North Carolina; § Duke University, Department of Biostatistics and Bioinformatics, Durham, North Carolina; ∥ Duke University, Department of Emergency Medicine, Durham, North Carolina; ¶ University of Mississippi Medical Center, Department of Emergency Medicine, Jackson, Mississippi

## Abstract

**Introduction:**

Pain is a major driver of visits to the emergency department (ED). Clinicians must consider not only the efficacy of treatment options but also subsequent healthcare utilization and patient-centered outcomes such as side effects from prescribed medications. Our goal in this study was to determine whether there was an association between acute pain treatment regimen (opioids, intranasal non-steroidal anti-inflammatory drugs [NSAIDs], or both) and unscheduled healthcare visits following ED discharge.

**Methods:**

This study was a secondary analysis of the Acute Management of Pain from the Emergency Department (AMPED) prospective, observational cohort study. We used Cox proportional hazards analysis to assess the relationship between treatment regimen and time to first unscheduled healthcare visit. Repeated measures logistic regression analyses were used to determine the relationship between treatment regimen and any unscheduled visits, and to evaluate whether this relationship was mediated by pain severity and/or medication side effects.

**Results:**

Of 831 total enrolled participants, 141 (16.9%) experienced an unplanned healthcare visit within five days of ED discharge. A majority of these visits happened one day after the ED visit. Those who were treated with intranasal NSAIDs only were less likely to have an unscheduled healthcare visit compared to those who received opioids only, with an adjusted odds ratio (AOR) of 0.63. The higher odds of unscheduled healthcare visits with opioids were mediated by both the presence of side effects and higher pain levels, with AORs of 2.24 and 1.33, respectively.

**Conclusion:**

Opioid treatment for acute pain is associated with increased unscheduled healthcare visits compared to those treated with intranasal ketorolac. This difference can be explained by higher levels of ongoing pain and greater medication side effects.

Population Health Research Capsule What do we already know about this issue?

*Wide variability in pain treatment in acute care settings is due in part to limited evidence on patient experiences related to choice of medication.*

What was the research question?

*What is the association between choice of acute pain treatment and unscheduled healthcare visits after ED discharge?*

What was the major finding of the study?

*Compared to opioids, NSAIDs had a lower adjusted odds ratio of 0.63 for post-ED unscheduled healthcare visits (P = 0.03, 95% CI 0.42–0.95, 33.3% of patients vs. 39.7%).*

How does this improve population health?

*Our study further underscores and contextualizes the wider complexity of the impact of treatment decisions (eg, medication side effects, comparative efficacy) on patient outcomes.*

## INTRODUCTION

Acute pain is the most frequent chief complaint for emergency department (ED) visits in the United States.^1^ Inadequate treatment of pain contributes to the evolution from acute to chronic pain, which can inflict excessive personal and economic burdens on patients.^2^ ^–^ ^4^ Outside the limited number of recommendations guiding treatment of a few specific acute conditions, such as lower back pain,^5^ there are no evidence-based guidelines that address the management of acute pain, resulting in wide variability in practice.^6^ ^–^ ^8^

In particular, the number of opioid prescriptions increased during the late 1990s to early 2010s, yet opioids were not found to correlate with improved pain-related outcomes or patient satisfaction.^9^ ^–^ ^11^ Moreover, the advent of the current opioid crisis and rise in opioid-related deaths motivated new guidelines discouraging the use of opioids for acute and chronic pain management.^12^ ^–^ ^14^ While opioid-prescribing rates subsequently began to decline from 21.5% of patients discharged from an ED in 2011 to 8.1% in 2019, this rate remains comparatively high.^15^ In addition, opioid reductions have not appeared to be matched by sufficient increases in non-opioid prescribing.^16^

In particular, ongoing concerns about the side effects of alternatives to opioids such as non-steroidal anti-inflammatory drugs (NSAID) have hindered their use.^17^ However, data shows that even in high-risk patients, those prescribed NSAIDs did not experience higher rates of serious adverse events.^18^ ^,^ ^19^ Specifically, intranasal ketorolac has been found to be an effective, well-tolerated, and satisfactory medication for adults presenting to the ED with acute pain, with only minor side effects such as nasal burning that resolves within several minutes.^20^ ^,^ ^21^ By comparison, the adverse effects of opioids outside opioid use disorder-related morbidity have received relatively little attention, with only recent data suggesting greater side effects with opioids than with NSAIDs.^19^ ^,^ ^22^ Thus, more studies are needed to better understand the risk-benefit comparison between opioids and NSAIDs for pain treatment.

Meanwhile, there has been a focus on preventing recurrent visits to the ED^23^ ^–^ ^25^ for pain as well as other complaints. Revisits substantially impact both the patient and healthcare system and often reflect unmet patient needs. Recent data suggests opioid prescriptions in the outpatient setting are associated with an increased number of subsequent healthcare visits.^26^ ^–^ ^29^ Unplanned visits can result in missed days of work,^30^ ^–^ ^32^ disrupting the daily lives of patients, as well as increased costs, often higher with the repeat than the initial ED visit.^33^ Failure to address the initial presenting problem or its sequelae, as well as lack of communication with patients regarding the therapeutic plan and recommended follow-up, contribute to ED recidivism.^32^ ^,^ ^34^ ^,^ ^35^ Emergency physicians must understand how the treatment they provide in the ED may affect downstream healthcare utilization to fully consider the impact of their care on both patients and healthcare systems.

In this secondary analysis, we sought to determine whether there was an association between treatment regimen (opioid only, intranasal NSAID only, or intranasal NSAID + opioid) following an ED visit for acute pain and subsequent daily healthcare utilization. We further characterized this association by whether medication side effects or ongoing pain severity mediated the effect on healthcare utilization.

## METHODS

### Study Setting and Population

This was a secondary analysis of data obtained from a multisite, prospective, observational cohort study, Acute Management of Pain from the Emergency Department (AMPED).^36^ The original study was approved by the institutional review boards at all enrollment sites. A convenience sample of adult participants ≥18 years old with acute musculoskeletal or visceral pain not requiring admission were recruited from 13 EDs in the US between September 2012–February 2014. Patients were prescribed one of three treatment regimens following ED discharge: NSAID (intranasal ketorolac) only; oral opioid only; or both (intranasal ketorolac with opioid as a rescue therapy). Treatment regimen was at the discretion of the treating clinician.

### Data Collection

Data collection included patient demographics, employment status, pain type (visceral or musculoskeletal) and location, pain scores (0–10 numeric rating scale) at ED triage and ED discharge, medications given during the ED visit, and discharge medication regimen. Patients were contacted daily for four days following ED discharge for follow-up outcomes including unplanned healthcare visits, medication use, daily highest and lowest pain scores, adverse events and symptoms, overall quality of life, and overall satisfaction with the prescribed pain medication. Details of the unplanned healthcare visits were not recorded, including reason for the visit.

### Statistical Analysis

All 831 patients were included for analysis. We used descriptive statistics to summarize patient demographics for those with or without unplanned healthcare visits after ED discharge. Categorical variables were reported as frequencies, and continuous variables were reported as median values with interquartile ranges (25^th^, 75^th^). Fewer than five patients had missing demographic data. The missing data on categorical variables were imputed to the highest frequency category, and continuous variables were imputed tomedian values.

We performed statistical analyses to investigate whether treatment regimen impacted the time to a subsequent unscheduled healthcare utilization following ED discharge and the number of any unscheduled visits, and to evaluate possible mediating factors. Those who received opioid-only treatments were used as the reference category for treatment regimen. We adjusted all models for age, gender, race, primary ED diagnosis, initial pain score, and discharge pain score. A two-tailed *P*-value of 0.05 was used for statistical significance. We performed all analyses using SAS 9.4 (SAS Institute Inc, Cary, NC).

To evaluate the relationship between treatment regimen and time to first unscheduled visit following ED discharge, we used a Cox proportional hazards model. Unscheduled visit (UV) data was collected over the four-day follow-up period. The first occurrence of an unscheduled visit (UV = YES) was used as the day of the unscheduled visit, regardless of UV = MISSING or UV = NO values in prior days. If the UV data was missing for all four days, the patient was censored at day 4. We generated a Kaplan-Meier curve of unscheduled visit with log-rank *P*-value. To evaluate the relationship between treatment regimen and any unscheduled visits following ED discharge, we used a repeated measures logistic regression model. Missing data on unscheduled visits over the four-day period were imputed as UV = NO value. Additionally, we performed a sensitivity analysis where all missing data on unscheduled visits were imputed as UV = YES value.

To evaluate for mediation of the association between treatment regimen and any unscheduled visits, we performed three separate repeated measures logistic regression models to assess the effect of 1) any medication side effect; 2) daily maximum pain scores; or 3) both side effects and pain scores.

## RESULTS

### Participant Characteristics

A total of 831 participants were included in the original AMPED study. As participants had to complete at least three of the four follow-up calls to be included in the original analysis, we did not exclude any patients from this secondary analysis. The distribution of participants in each discharge treatment regimen group was uneven in the original study, with combination therapy (intranasal ketorolac with opioid rescue) being the least prescribed regimen.^36^ As reported in the parent study, characteristics of participants in each discharge treatment group were comparable, except that participants in the opioid-only group were slightly older and more likely to have a chronic pain history; participants who had fractures or visceral pain were more likely to receive opioids as part of their regimen; and participants in the intranasal ketorolac-only group had slightly lower average pain scores at initial ED presentation.^36^ Overall, 141 (16.9%) participants had at least one unscheduled visit over the four-day follow-up time period. Baseline and demographic characteristics of participants in this sub-analysis are shown by attendance to unscheduled visits (none vs at least one visit) in Table 1.

**Table 1.** Baseline characteristics of the AMPED^*^ participants by unscheduled visits.

			Unscheduled visit	
	Level	Overall (N = 831)	No unscheduled visit (n = 690)	At least one unscheduled visit (n = 141)
Age (median, IQR)		37.0 (27.0, 48.0)	36.0 (27.0, 49.0)	39.0 (31.0, 48.0)
	Missing, n (%)	1 (0.1)	1 (0.1)	0 (0.0)
Treatment regimen, n (%)	Intranasal ketorolac only	353 (42.5)	306 (44.3)	47 (33.3)
	Intranasal ketorolac + opioid	201 (24.2)	163 (23.6)	38 (27.0)
	Opioid Only	277 (33.3)	221 (32.0)	56 (39.7)
	Missing, n (%)	0 (0.0)	0 (0.0)	0 (0.0)
Employment status, n (%)	Other	92 (11.1)	78 (11.3)	14 (9.9)
	Unemployed	128 (15.4)	109 (15.8)	19 (13.5)
	Part time	129 (15.5)	103 (14.9)	26 (18.4)
	Full time	481 (57.9)	399 (57.8)	82 (58.2)
	Missing, n (%)	1 (0.1)	1 (0.1)	0 (0.0)
Gender, n (%)	Male	434 (52.2)	373 (54.1)	61 (43.3)
	Female	396 (47.7)	316 (45.8)	80 (56.7)
	Missing, n (%)	1 (0.1)	1 (0.1)	0 (0.0)
Race, n (%)	Other	169 (20.3)	148 (21.4)	21 (14.9)
	Caucasian	251 (30.2)	193 (28.0)	58 (41.1)
	Black	407 (49.0)	345 (50.0)	62 (44.0)
	Missing, n (%)	4 (0.5)	4 (0.6)	0 (0.0)
Primary ED diagnosis, n (%)	Musculoskeletal	707 (85.1)	593 (85.9)	114 (80.9)
	Visceral	124 (14.9)	97 (14.1)	27 (19.1)
	Missing, n (%)	0 (0.0)	0 (0.0)	0 (0.0)
Pain score in ED (median, IQR)		8.0 (7.0, 10.0)	8.0 (7.0, 10.0)	8.0 (7.0, 10.0)
	Missing, n (%)	1 (0.1)	1 (0.1)	0 (0.0)
Pain score at discharge (median, IQR)		5.0 (3.0, 8.0)	5.0 (3.0, 8.0)	5.0 (3.0, 8.0)
	Missing, n (%)	1 (0.1)	1 (0.1)	0 (0.0)
4-day any side effect, n (%)	Yes	621 (74.7)	493 (71.4)	128 (90.8)
	No	210 (25.3)	197 (28.6)	13 (9.2)
	Missing, n (%)	0 (0.0)	0 (0.0)	0 (0.0)
4-day nausea, n (%)	Yes	284 (34.2)	215 (31.2)	69 (48.9)
	No	542 (65.2)	470 (68.1)	72 (51.1)
	Missing, n (%)	5 (0.6)	5 (0.7)	0 (0.0)
4-day vomited, n (%)	Yes	82 (9.9)	57 (8.3)	25 (17.7)
	No	744 (89.5)	628 (91.0)	116 (82.3)
	Missing, n (%)	5 (0.6)	5 (0.7)	0 (0.0)
4-day constipation, n (%)	Yes	272 (32.7)	215 (31.2)	57 (40.4)
	No	554 (66.7)	470 (68.1)	84 (59.6)
	Missing, n (%)	5 (0.6)	5 (0.7)	0 (0.0)
4-day nasal irritation, n (%)	Yes	208 (25.0)	168 (24.3)	40 (28.4)
	No	618 (74.4)	517 (74.9)	101 (71.6)
	Missing, n (%)	5 (0.6)	5 (0.7)	0 (0.0)
4-day rash/hives, n (%)	Yes	33 (4.0)	17 (2.5)	16 (11.3)
	No	793 (95.4)	668 (96.8)	125 (88.7)
	Missing, n (%)	5 (0.6)	5 (0.7)	0 (0.0)
4-day abdominal pain, n (%)	Yes	167 (20.1)	122 (17.7)	45 (31.9)
	No	659 (79.3)	563 (81.6)	96 (68.1)
	Missing, n (%)	5 (0.6)	5 (0.7)	0 (0.0)
4-day drowsiness, n (%)	Yes	412 (49.6)	323 (46.8)	89 (63.1)
	No	414 (49.8)	362 (52.5)	52 (36.9)
	Missing, n (%)	5 (0.6)	5 (0.7)	0 (0.0)

* *AMPED*, Acute Pain Management from the Emergency Department; *IQR*, interquartile range; *ED*, emergency department.

### Model Results

**Figure 1.**
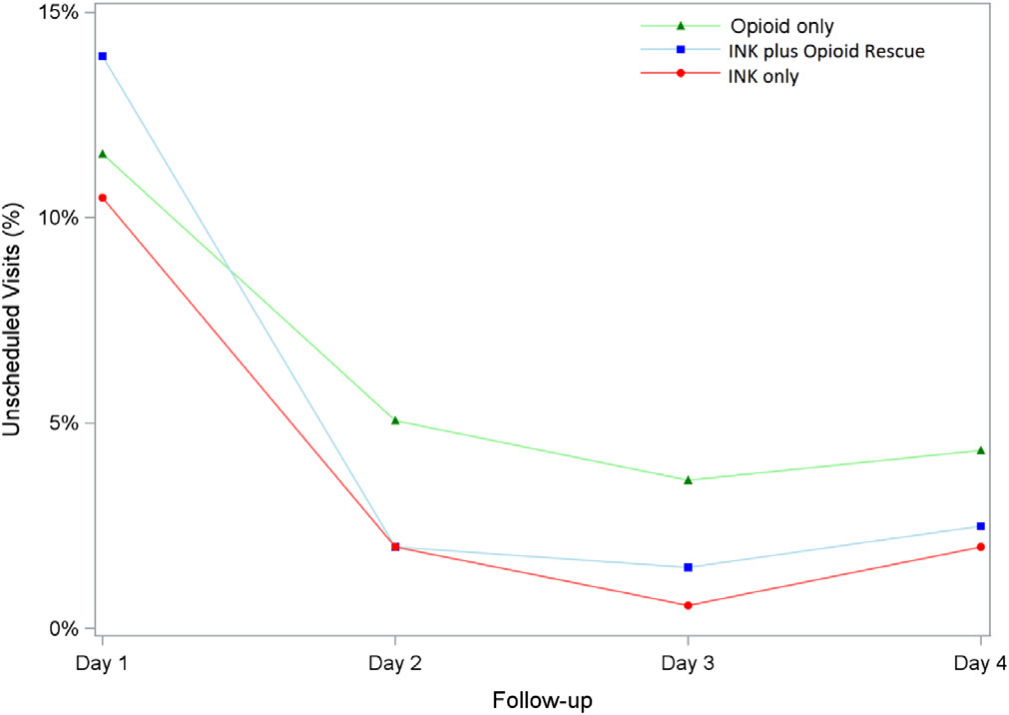
Daily unscheduled visit rates by discharge treatment regimen.

**Figure 2.**
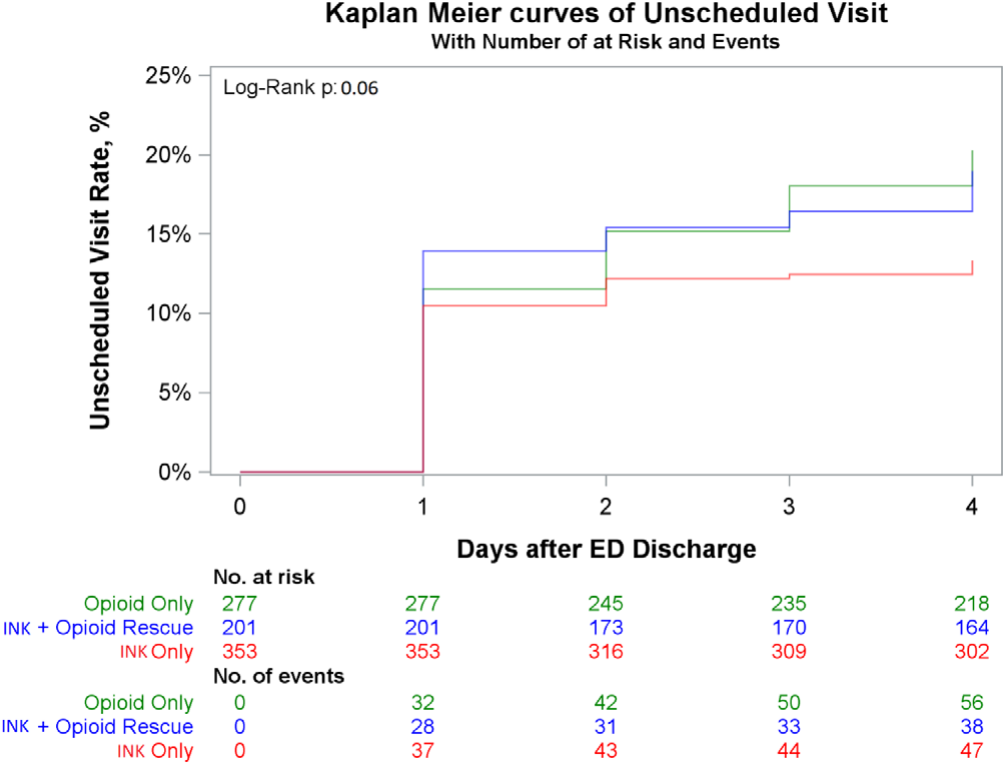
Kaplan-Meier curve of unscheduled visits for treatment regimen with number of events and at risk.

Across all treatment regimens, the highest number of unscheduled visits occurred on the first day of the four-day follow-up period (Figure 1). However, after adjustment of the hazard ratios (*P* > 0.05), there did not appear to be a statistically significant association between pain medication prescribed and time to first unscheduled visit (Table 2). Kaplan-Meier curve of unscheduled visit with log-rank *P*-value is provided in Figure 2.

**Table 2.** Unadjusted and adjusted hazard ratios of time to first unscheduled visit.

Outcome	Parameter	Unadjusted HR	Unadjusted *P-*value	Adjusted HR^*^	Adjusted *P*-value
Unscheduled visits	Opioid only (reference)				
	Intranasal ketorolac + opioid rescue	0.9 (0.6, 1.4)	0.75	0.9 (0.6–1.4)	0.74
	Intranasal ketorolac only	0.7 (0.4, 1.0)	0.03	0.7 (0.5–1.0)	0.07

HR, hazard ratios (95% confidence intervals).

* Adjusted for age, gender, race, primary ED diagnosis, pain score prior to treatment and pain score at discharge.

When we consider all unscheduled visits over the four-day follow-up period, participants who were treated with intranasal ketorolac only were less likely to have an unscheduled healthcare visit compared to those who received opioids only, with an adjusted odds ratio of 0.63 (Table 3). The sensitivity analysis showed that model effects were not meaningfully altered by missing data. The mediation analysis demonstrated that the association between treatment regimen and unscheduled healthcare visits is completely explained by both the presence of side effects and the severity of pain, with odds ratios of 2.24 and 1.33, respectively (*P* = <0.001), and side effects contributing the larger impact (Table 4).

**Table 3.** Unadjusted and adjusted odds ratios of any unscheduled visit.

Outcome	Parameter	Unadjusted OR	Unadjusted *P*-value	Adjusted OR^*^	Adjusted *P*-value
Unscheduled visits	Opioid only (reference)				
	Intranasal ketorolac + opioid rescue	0.81 (0.53, 1.25)	0.348	0.79 (0.51–1.22)	0.29
	Intranasal ketorolac only	0.60 (0.41, 0.89)	0.012	0.63 (0.42–0.95)	0.03

OR, odds ratios (95% confidence intervals).

* Adjusted for age, gender, race, primary ED diagnosis, pain score prior to treatment and pain score at discharge.

**Table 4.** Odds ratio for mediators – any side effect and maximum pain score.

Outcome	Parameter	Odds ratio	*P-*value
Unscheduled visits	Any side effect	2.24 (1.55, 3.24)	<.001
	Maximum pain score	1.33 (1.22, 1.44)	<.001
	Opioid only (reference)		
	Intranasal ketorolac + opioid rescue	1.01 (0.65, 1.57)	0.95
	Intranasal ketorolac only	0.96 (0.64, 1.45)	0.86

## DISCUSSION

In this study evaluating the relationship between treatment regimens (opioids, rapidly acting intranasal NSAIDs, or both) following an ED visit for acute pain and subsequent daily healthcare utilization, we found that treatment with opioids only was associated with increased subsequent unscheduled healthcare visits during the immediate post-ED discharge period compared to treatment with intranasal ketorolac. Similar associations have been reported previously between opioid treatment for chronic pain and increased frequency of ED visits, opioid prescriptions and recurrent ED visits for lower back pain, as well as opioid use and increased healthcare utilization following elective spine surgery.^26^ ^–^ ^29^ Despite this recent attention on the relationship between treatment choice and healthcare utilization, there is a disconcerting paucity of literature investigating possible mediating factors. In particular, several recent systematic reviews have shown both lower pain treatment efficacy and higher rates of adverse events with opioids compared with NSAIDs, emphasizing the importance of these factors when weighing the risks and benefits of treatment choice for acute pain management.^19^ ^,^ ^22^ ^,^ ^37^

Our study is an important contribution to the literature, as it is the first to our knowledge to demonstrate that the association between pain treatment regimens and subsequent healthcare utilization post-ED discharge is mediated by both the presence of medication side effects and ongoing, poorly controlled pain. These findings highlight additional risks and benefits related to these acute pain treatment regimens that may impact patient outcomes. Considering previous research demonstrating the greater financial burden of recurrent healthcare utilization on the patient compared to the initial visit^33^ as well as the reasons patients return to the ED—which range from lack of symptomatic improvement to additional questions or concerns^32^ ^,^ ^34^ ^,^ ^35^—our study serves to further contextualize and demonstrate the wider complexity of the impact of treatment decisions on patient outcomes. Thus, when choosing a pain management plan, it is important to consider the impact it may have on multiple aspects of the patient’s quality of life in conjunction with treatment efficacy.

## LIMITATIONS

Study limitations include that this was a secondary analysis of observational data and, therefore, not all potential confounders may have been measured or controlled for. Additionally, the study was not randomized, which may had led to selection bias with confounding factors that impacted choice of treatment regimen, which may have affected the likelihood of unscheduled visits. Further, the data collection occurred between the years 2012–2014, which may limit the applicability of these findings, as pain treatment regimens and opioid use have since evolved.

Despite the fact that overall rate of opioid prescriptions issued at discharge from the ED decreased from 14.6% in 2017 to 8.1% in 2020, there are still a large overall number of people who are receiving opioids.^15^ In addition, prescribing patterns should not change the underlying side-effect profile or their inherent associations with unscheduled visits. It is also important to note that while our findings support a strong association between treatment regimen and unscheduled healthcare visits mediated by the presence of side effects and degree of pain relief, the reasons for unscheduled visits were not confirmed by patient report. Thus, it is possible that patients sought healthcare for reasons unrelated to their acute pain, current treatment plan, or side effects. Finally, the treatment regimen was not standardized across all groups apart from the intranasal ketorolac; thus, the reported side effects and complications may also be related to differences in opioid type or dosing.

## CONCLUSION

Outpatient treatment with opioids only for acute pain after an ED visit is associated with increased unscheduled healthcare visits compared to those treated with intranasal NSAIDs alone. This difference can be explained by higher levels of ongoing pain and the presence of medication side effects. Understanding the impact that pain medication choice has on recurrent healthcare utilization and the factors that mediate this relationship adds to the body of knowledge regarding risks and benefits of these treatments, allowing emergency physicians to make better informed decisions.
